# Three-phase material mapping with incomplete X-ray diffraction spectral information

**DOI:** 10.1107/S160057672300331X

**Published:** 2023-05-23

**Authors:** Xuyang Chang, Karine Lavernhe-Taillard, Stéphane Roux, Olivier Hubert

**Affiliations:** a Université Paris-Saclay/CentraleSupélec/ENS Paris-Saclay/CNRS, LMPS – Laboratoire de Mécanique Paris-Saclay, F-91190, Gif-sur-Yvette, France; Tohoku University, Japan

**Keywords:** X-ray diffraction, shape-memory alloys, proper orthogonal decomposition

## Abstract

An equiatomic nickel–titanium shape-memory alloy specimen subjected to a uniaxial tensile load undergoes a two-step phase transformation under stress, from austenite (A) to a rhombohedral phase (R) and further to martensite (M) variants. The pseudo-elasticity that goes accompanies the phase transformation induces spatial inhomogeneity. To unravel the spatial distribution of the phases, *in situ* X-ray diffraction analyses are performed while the sample is under tensile load. However, the diffraction spectra of the R phase, as well as the extent of potential martensite detwinning, are not known. A novel algorithm, based on a proper orthogonal decomposition and incorporating inequality constraints, is proposed in order to map out the different phases and simultaneously yield the missing diffraction spectral information. An experimental case study illustrates the methodology.

## Abstract 2

A novel algorithm, based on a proper orthogonal decomposition and incorporating inequality constraints, is proposed to map out the different phases of a heterogenous material and simultaneously yield missing diffraction spectral information. An experimental case study of a three-phase NiTi shape-memory alloy under tensile loading illustrates the presented methodology.

## 1. Introduction

X-ray diffraction (XRD) is a popular non-destructive qualitative and quantitative technique aimed at characterizing crystal lattice parameters (Drickamer *et al.*, 1967 ▸), local strain (Gailhanou *et al.*, 2007 ▸), microstructure evolution (Oliveira *et al.*, 2022 ▸) or phase constituent proportions (Peng *et al.*, 2005 ▸) from analysed specimens (*e.g.* metals, polymers and ceramics). Although XRD has been primarily emphasized as an efficient tool for qualitative analyses, it is often used to perform quantitative measurements of the phase concentrations within a material. The Rietveld refinement method (McCusker *et al.*, 1999 ▸) is generally applied to conduct quantitative analysis of XRD patterns, but it requires the diffraction profiles for all possible phase constituents to be collected appropriately during the preparation stage, so that the individual components can be adequately identified afterwards. From a practical point of view, this preparation is rather demanding for (complex) heterogeneous specimens. Moreover, the preferred orientation effects of XRD measurement (Dickson, 1969 ▸; Campbell Roberts *et al.*, 2002 ▸) are extremely difficult to deal with experimentally.

A metallic specimen with a pronounced preferred orientation – especially with a flat-plate geometry – may exhibit a strong {*hkl*} intensity dependency when compared with theoretical powder diffraction patterns. Although many Rietveld refinement programs allow for the identification of an artificial preferred orientation parameter with respect to a specific crystallographic vector based on either the March–Dollase model (Dollase, 1986 ▸) or the generalized spherical harmonic model (Sitepu *et al.*, 2005 ▸), this remains a crude approximation when assessing heterogeneous multi-phase specimens.

In this work, a proper orthogonal decomposition (POD) algorithm, suitably extended to incorporate inequality constraints such as positivity (and referred to as positive-POD or p-POD), is proposed to circumvent the aforementioned challenges: For all phase constituents whose diffraction profiles are experimentally available (denoted ‘known constituents’), the POD technique (Chatterjee, 2000 ▸) is first applied to construct the experimental diffraction spectrum while taking the preferred orientation effect into account. Then, by enforcing positivity constraints, the phase concentrations for the known constituents can be estimated through a quadratic minimization with convex positive constraints using the sub-gradient projection algorithm (Boyd *et al.*, 2003 ▸). Finally, the phase concentration and experimental diffraction data for the unknown constituents can be obtained.

The performance of the proposed algorithm is illustrated using a strip-shaped specimen made of equiatomic nickel–titanium shape-memory alloy (NiTinol) subjected to a uniaxial tensile load. Under stress, nickel–titanium alloys are frequently reported to undergo a two-step martensitic phase transformation in the form of strain localization bands at ambient temperature [from austenite (A) to rhombohedral (R) and further to martensite (M)] (Miyai *et al.*, 2006 ▸; Halani *et al.*, 2013 ▸; Stebner *et al.*, 2015 ▸).

At a given load, the XRD profiles are recorded, scanning through the specimen along the tensile direction, *i.e.* across the strain localization bands, so as to elucidate the on-going phase transformation(s) from the progressive changes in the diffraction spectra. For NiTi shape-memory alloys, depending on the forming process and chemical composition, when subjected to mechanical loads the R phase sometimes appears as an intermediate phase in a two-step phase transformation. It usually co-exists with austenite at the macroscopic scale (whatever the stress or thermal load), which impedes the measurement of its individual diffraction spectrum. In contrast, it is possible to find specific conditions for which pure austenite or martensite phases exist in the specimen.

Moreover, the formation of strain localization bands is a macroscopic outcome of the ‘martensite detwinning’ process (Ng & Sun, 2006 ▸) (also known as ‘martensite variants selection’), resulting in a pronounced preferred orientation effect.

The missing R-phase diffraction pattern and the un­determined preferred orientation of martensite variants prevents the Rietveld refinement method from achieving any comprehensive results. To overcome this limitation, the proposed p-POD algorithm permits the estimation of the concentration of different phases along the sample and provides an estimated R-phase diffraction spectrum. The proposed method is extremely versatile, since it requires neither complete knowledge of the diffraction data for all constituents nor challenging experimental processing to remove the signature of preferred orientations in the specimen.

The paper is organized as follows. Section 2 presents the combined *in situ* XRD and digital image correlation (DIC) measurement setups and the associated strain fields and raw diffraction spectra acquired during 1D tensile loading. The Rietveld processing method is recalled briefly in Section 3. Section 4 introduces the positive POD algorithm to conduct phase field reconstruction. Section 5 applies the proposed algorithm to the spectra of NiTinol recorded in scans along the tensile axis at different stages of loading. Section 6 draws some conclusions.

## 2. Equiatomic NiTinol under uniaxial tensile loading

### 2.1. Tested specimen

**Figure 1:**
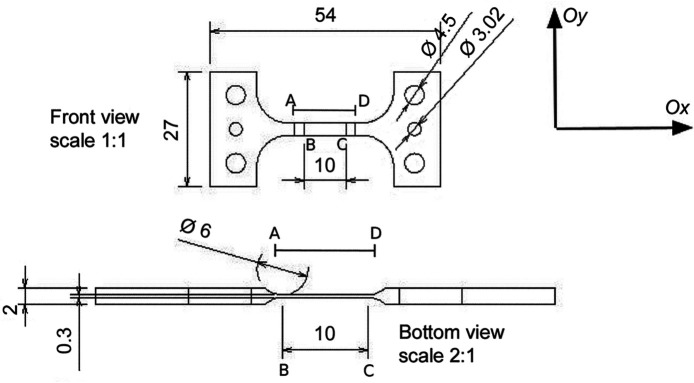
The geometry of the specimen for the uniaxial tensile test. The effective zone for XRD measurement is between *A* and *D*, of length *L*_*AD*_ = 14 mm.

A specimen of quasi-equiatomic Ni–Ti alloy (NiTinol) (thin parallelepipedic central zone of length *L*_*AD*_ = 14 mm, width *l* = 3 mm and thickness *h* = 0.3 mm) was positioned (Fig. 1 ▸) in a mechanical testing machine located within an XRD chamber. The specimen surface was speckled with white paint to provide enough contrast for DIC analyses to measure the displacement field under load. Attention was paid to ensuring that the paint did not affect the diffraction pattern over the range of diffraction angles of interest in the following.

### 2.2. Experimental setups

**Figure 2:**
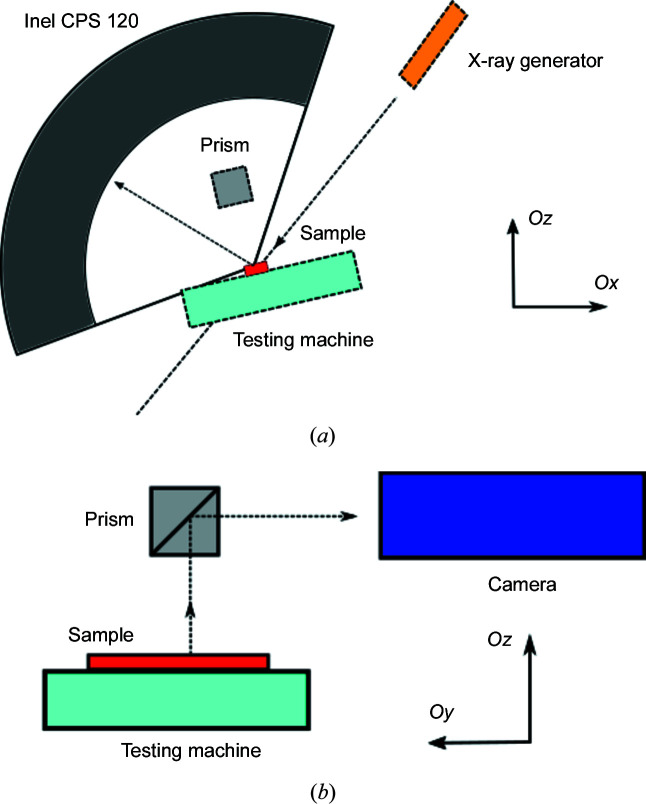
Schematic diagrams of the *in situ* combined multi-field measurement utilizing XRD and digital image correlation. (*a*) The angular XRD measurement setup in the *Oxz* plane. (*b*) An illustration of the DIC setup in the *Oyz* plane.

As shown in Figs. 2 ▸ and 3 ▸, a wide-angle X-ray diffractometer equipped with a conventional X-ray source (cobalt *K*α with a wavelength λ = 1.79 Å) and a curved detector (Inel CPS-120) was used to measure the XRD spectrum along diffraction angles 2θ varying in the range 20 < 2θ < 140°. An Fe filter was used to suppress the contribution of Co *K*β to the X-ray radiation.

To limit any cross-influence between the two setups, a prism was used to redirect the image of the specimen surface towards a visible-light camera (Camera 1). A displacement-controlled 1D tensile test was carried out at room temperature (*T* = 300 K) with a loading speed 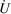 = 1 µm s^−1^ (corresponding to a longitudinal strain rate 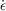 = 10^−4^ s^−1^). An initial XRD scan was conducted in the stress-free configuration. The mechanical test was then interrupted three times along the loading stage, during which the displacement was held constant (*U* = 0.3, 0.4 and 0.6 mm), and a similar holding time was considered during unloading (*U* = 0.3 mm), to perform the XRD measurement along a longitudinal profile (referred to as a ‘scan’ in the following). The corresponding stress/strain curve is reported in Fig. 4 ▸(*a*). The DIC-measured deviatoric strain field and strain rate are plotted in Fig. 4 ▸(*b*). Two localization bands are formed, and the region where the strain is above 5% corresponds to the region which has transformed into martensite. The strain-rate field highlights the transformation front. The temperature elevation due to the latent heat released in the phase transformation prevents the propagation of a single transformation front, and hence several bands are observed in the tested specimen.

The geometry of the specimen is given in Fig. 1 ▸. The designed region of interest is 10 mm long between points *B* and *C*, with *B* and *C* marked with very shallow landmarks. However, points *B* and *C* could no longer be seen during the experiment for both DIC and XRD measurements after spraying the speckle patterns onto the specimen. As a result, the effective XRD region length was 14 mm long, between points *A* (*x* = 0) and *D* (*x* = 14). At each of the four interruptions (denoted Steps 0–3) [shown as red marks in Fig. 4 ▸(*a*)], XRD spectra were recorded, scanning along the load axis in the central part of the Ni–Ti specimen (corresponding to spatial scanning coordinates 0 mm ≤ *y* ≤ 14 mm).

### 2.3. Spatial spectra

The raw spectra collected at the four interruption stages are plotted in Fig. 5 ▸. For the sake of readability, here and in the following an offset proportional to the *y* coordinate of the studied spot is added to the spectra so that they do not overlap.

#### 2.3.1. Masking and clipping

**Figure 3:**
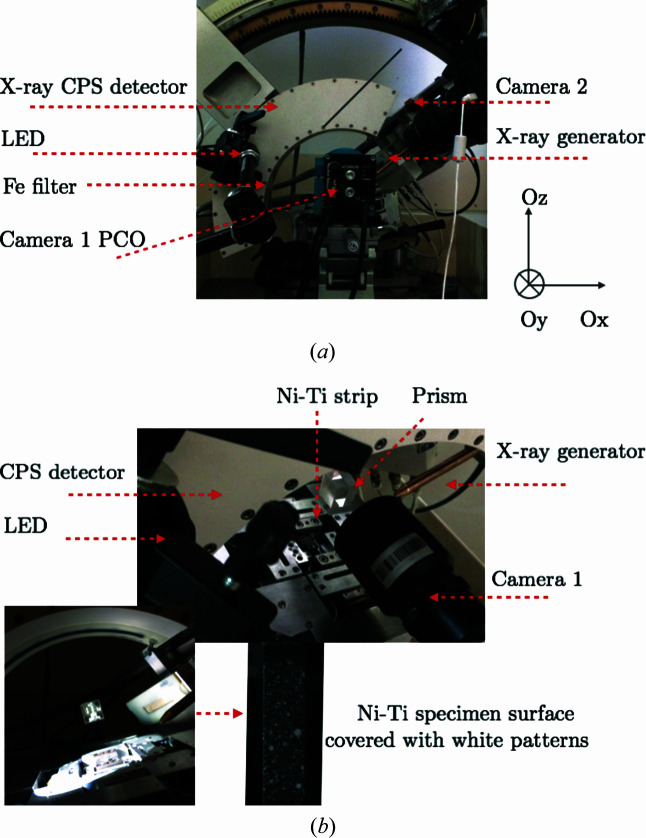
Different views of the *in situ* combined XRD and DIC measurement setup.

As indicated in Fig. 5 ▸, some angular intervals of the recorded spectra are not reliable:

(i) The contribution of Co *K*β to the experimental XRD should be excluded to grant us a single dominant wavelength of λ to facilitate the indexing of diffraction peaks using diffraction theory.

(ii) The Fe filter gives rise to a double absorption in the range of diffraction angles 62 ≤ 2θ < 65°.

(iii) The optical prism obscures the X-ray detector for 2θ ≥ 100° during Steps 1–3 (during Step 0 the prism was not yet installed), as shown in Fig. 3 ▸.

In order to perform the peak indexing of the diffractograms properly, masking all three of these regions is mandatory. Consequently, all diffraction signals shaded in grey as shown in Fig. 5 ▸, within the corresponding ranges of diffraction angles (that is [44°, 46°] ∪ [62°, 65°] ∪ [100°, 140°]), are omitted from the analysis throughout this paper.

#### 2.3.2. Qualitative analysis

The crystallography of NiTinol alloys has been extensively investigated. With the published crystallographic information [A and M (Bhattacharya, 2003 ▸), and R (Zhang & Sehitoglu, 2004 ▸)], it is possible to use powder diffraction theory (Cullity, 1956 ▸) to construct theoretical diffractograms (as illustrated in Fig. 6 ▸) and provide qualitative characterization of the phase constituents present at the four different stages.

Step 0. In the ambient load-free state (σ = 0 MPa, *T* = 300 K), NiTinol is usually considered as pure austenite. The {*hkl*} diffraction peaks at different spatial coordinates in Fig. 5 ▸(*a*) at 2θ = 49 and 92° correspond to A{110} and A{211}, respectively, which are consistent with the theoretical XRD diffractograms. The {200} peak is not visible in the experimental spectra because of the pronounced crystallographic texture of this laminated material (Chang *et al.*, 2020 ▸). Therefore, at this stage, the collected experimental diffraction spectra are taken as the data profile for pure austenite.

**Figure 4:**
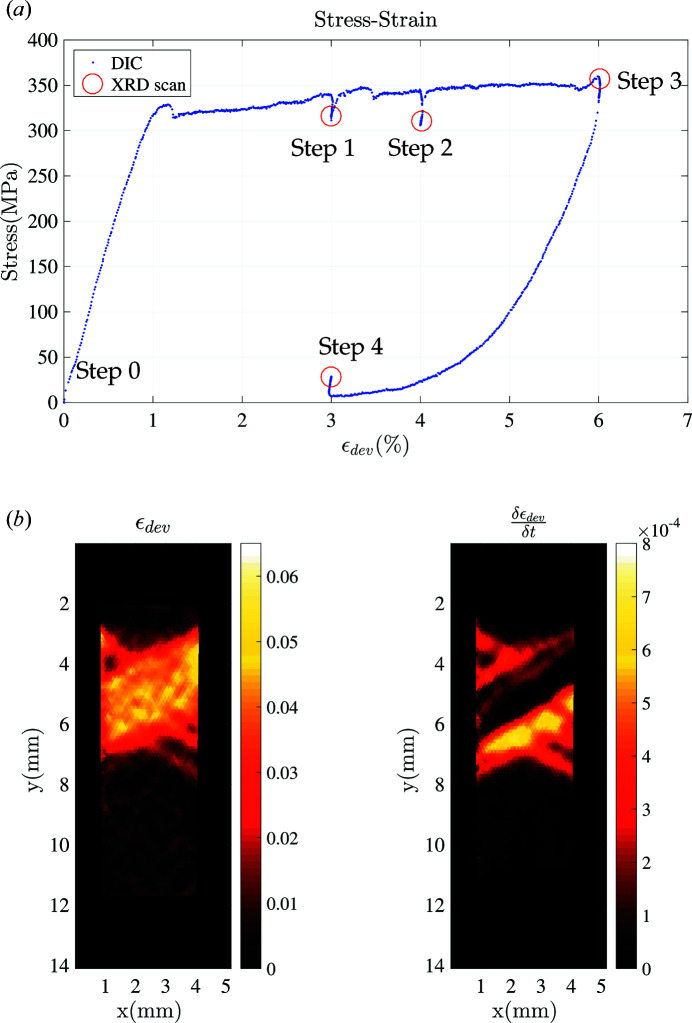
(*a*) A stress–strain curve during the 1D tensile loading. The positions where the XRD scans were recorded are shown as red circles. (*b*) The (Eulerian) deviatoric strain (left) and strain rate (right) fields at the first loading stage (scan performed at Step 1), as measured by DIC, appear to be strongly heterogeneous.

**Figure 5:**
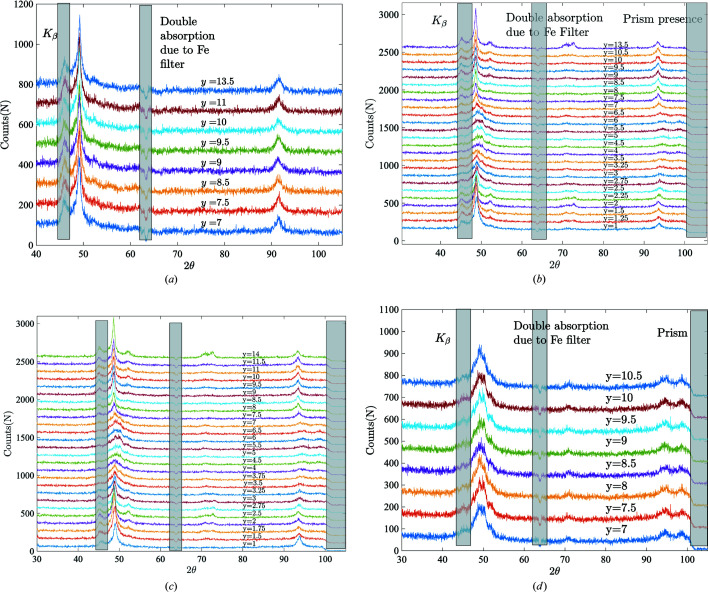
Diffraction spectra at different spatial coordinates. (*a*) Step 0, the stress-free state. (*b*), (*c*) Steps 1–2 (intermediate stages), on-going martensitic transformation inside the region of interest. (*d*) Step 3 (maximum displacement), the central zone of the sample is fully transformed into martensite.

**Figure 6:**
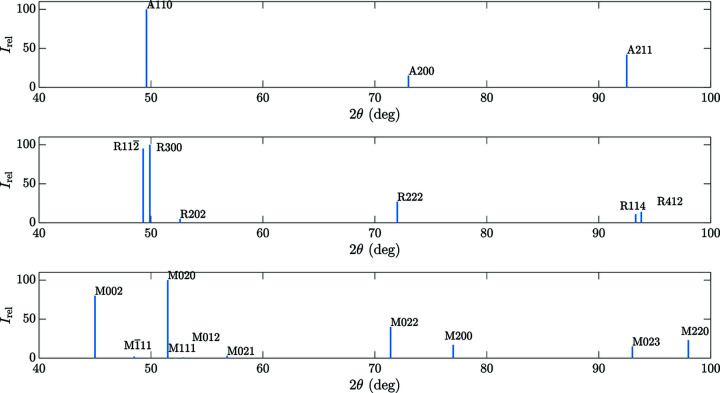
Theoretical XRD profiles (integrated intensities) for (top) austenite, (middle) R phase and (bottom) martensite.

Steps 1 and 2. When comparing the diffractograms at different spatial locations as illustrated in Figs. 5 ▸(*b*) and 5 ▸(*c*), pronounced differences can be observed. For example, focusing on the range 48 ≤ 2θ ≤ 52°, a large shift in the peak position and in its broadening characterized by its full width at mid-height (FWMH) can be seen along the sample axis. For all spectra acquired in the range 2 ≤ *y* ≤ 4 mm [within the high-strain band as illustrated in Fig. 4 ▸(*b*)] the spectra appear as pure martensite, characterized by the typical double martensite peaks in the range 90 ≤ 2θ ≤ 100°. Outside the transformed region, the co-existence of A and R phases can be verified by the secondary peak at 2θ = 51°, which can be unambiguously attributed to the R {202} crystal lattice diffraction plane.

Step 3. Experimental pure martensite diffraction spectra can be collected when the external loading has reached the end of the stress–strain transformation plateau, at point ‘Step 3’ marked with a red circle in Fig. 4 ▸. The corresponding diffractogram in Fig. 5 ▸(*d*) can be considered as pure martensite with a typical double martensite peak in the region 90 ≤ 2θ ≤ 100°.

From Fig. 5 ▸, a series of raw spectra **D**(2θ, *y*) are obtained, each of which is composed of the three phases of NiTinol: A, M and R. For the proper identification of these phases, it is possible to prepare the specimen to obtain pure A and M phases with either a load-free state at high temperature (austenite) or under load after a full transformation (martensite), with a representative anisotropy along the tensile axis due to the selection of variants. However, this is not the case for the R phase: in a previous study (Chang *et al.*, 2020 ▸), by conducting differential scanning calorimetry (DSC) over the NiTinol specimen, it was shown that the R phase is present only in the intermediate stages and never uniformly.

With careful sequential Rietveld refinement, thermally induced martensite detwinning diffraction profiles and associated concentrations can be accurately estimated, as reported by Oliveira *et al.* (2021 ▸). However, in the present case, the combination of missing knowledge of the R diffraction profiles and possible preferred orientation effects due to martensite detwinning makes it very difficult to use the Rietveld refinement method to conduct any reliable quantitative analysis. To highlight these difficulties better, and how we propose to circumvent them with the positive POD algorithm, the Rietveld method and its limitations are briefly recalled in the following section.

## 3. Rietveld method

The original presentation of the Rietveld method (Rietveld, 1969 ▸) is followed in the discussion below.

### 3.1. Integrated intensities and estimated diffraction profile

For Bragg diffraction peaks, the integrated intensities *I*_{*hkl*}_ can be written as 

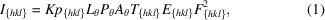

where *K* is a scale factor, *p*_{*hkl*}_ is a multiplicity factor to account for symmetry in the reciprocal lattice, *L*_θ_, *P*_θ_ and *A*_θ_ represent, respectively, the Lorentz, polarization and absorption factors, *T*_{*hkl*}_ is the preferred orientation factor, *E*_{*hkl*}_ designates the extinction factor, and *F*_{*hkl*}_ is the structure factor.

Various effects generate a Gaussian-like broadening of each peak, so that the estimated diffraction profile for a given pure phase α can be approximated as 

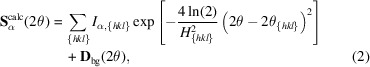

where *H*_{*hkl*}_ represents the full width at half-maximum (FWHM) for a given {*hkl*} peak and **D**_bg_(2θ) represents the background of the XRD signal.

### 3.2. Rietveld refinement

Several assumptions are made here: diffraction profiles between different phase constituents follow a natural mixture law, and other XRD parameters are known (*e.g.* background, FWHM, asymmetry parameters, unit-cell dimensions, preferred orientation *etc.*). Rietveld refinement aims to determine the phase concentration 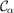 through a nonlinear least-squares fitting, 

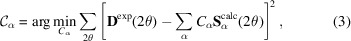

where **D** ^exp^ represents the experimental diffraction profile.

Despite the widespread usage of Rietveld refinement in XRD characterization, three major limitations need to be specifically addressed.

(i) Noise. The quadratic cost function implemented in Rietveld refinement rests upon the assumption that the noise in the XRD measurement follows a Gaussian distribution with a uniform variance (in 2θ) for 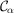 to be optimal. However, for X-ray detectors, it is commonly reported that noise follows a Poisson distribution. In such a case, the chosen quadratic cost function in Rietveld refinement is not ‘wrong’ but neither is it optimal. Hence the nature of the measured noise needs to be characterized first and the cost function needs to be adapted accordingly, with a weight proportional to the inverse intensity.

(ii) Correlated parameter fitting. To the best of the authors’ knowledge, most commercial program codes using Rietveld refinement require a sequence of parameter refinement (background, unit-cell crystal parameters, asymmetry parameters, preferred texture factor *etc.*) to reach the sought phase concentration. Therefore, when dealing with data from a mixture of different phases with a pronounced texture preference, the nonlinear least-squares fitting is prone to secondary minimum trapping, and the set of correlated parameters (concentration, preferred orientation) are ill-estimated. Thus, simplifying the Rietveld refinement protocol and determining the phase concentration with fewer model parameters is appealing.

(iii) The effect of preferred orientation. This question is discussed further in Section 3.3.

### 3.3. The effect of preferred orientation

In this section, in addressing the role of preferred orientation for a single phase α, it is assumed that other parameters remain constant through the entire experiment.

#### 3.3.1. Case 1: a single crystal

If the crystal lattice orientation of the phase is unique and represented using a rotation quaternion denoted *n*_1_, the diffraction profile is denoted 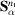 , which can be seen as the set of intensities of the different {*hkl*} Bragg diffraction peaks.

For a single crystal having a different crystal orientation, say *n*_2_, its diffraction profile is related to the former from a matrix transformation *T*(*n*_1_, *n*_2_) such that 

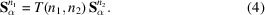




#### 3.3.2. Case 2: a single crystallographic phase with a strong texture

When a strong texture is present in the tested specimen, it is possible to use a reduced basis (*n*_1_, *n*_2_) to represent the distribution of possible crystal lattice orientations. The concentration of any experimental diffraction profile from phase α can be approximated with the corresponding diffraction profiles 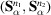 ,

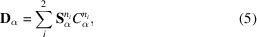




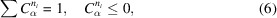




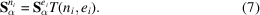




#### 3.3.3. Case 3: a single crystallographic phase but following a statistical distribution of orientations for multiple grains

It must be recalled that only three directions (for example, one of the most conventional choices is the 〈100〉, 〈110〉, 〈111〉 orientation used for pole figures) are required to map the entire crystal orientation of the specimen. Consequently, as a natural expansion of equation (4), any experimental diffraction profile of the polycrystal can be expressed as a linear combination, 

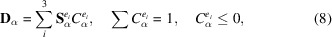

where 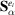 and 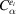 represent, respectively, the XRD profile following a given direction of the crystallographic lattice plane *e*_*i*_ and its associated concentration. {*e*_1_, *e*_2_, *e*_3_} represents any basis of the diffracting crystal plane orientation.

Several remarks are to be made here:

(i) Equation (9) can be considered as the generalized formulation of the experimental diffraction profiles of a pure phase (either a single crystal or a polycrystal with a pronounced texture preference are particular cases).

(ii) Hereinafter, 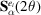 is used to represent the XRD profile for a single phase with a particular orientation *e*_*i*_ (after convolution accounting for instrumental acquisition). In this respect, other model parameters are no longer to be identified independently for phase concentration determination. Thus, the number of unknowns in the refinement is drastically reduced.

(iii) When the preferred orientation effect is present in the tested sample, it requires at least two (and at most three) sets of preferred orientation factors; hence two or three diffraction profiles are needed for the same phase to guarantee a trustworthy phase concentration estimation. The preferred orientation correction implemented in the Rietveld refinement method is a first-order correction considering the principal crystal lattice orientation

(iv) The non-uniqueness of the (reduced) basis *e*_*i*_ is very beneficial. Taking a highly textured specimen as an example (Appendix *A* [App appa]), one could select any reduced basis (*n*_1_, *n*_2_) and its corresponding diffraction profiles 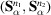 to calculate the corresponding amplitudes 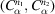 .

The positive POD algorithm is introduced in the following section, and a proof of concept is given to construct a reduced basis from experimental data.

## 4. p-POD algorithm

The analysis consists of five steps:

(i) Correction of background contribution.

(ii) Evaluation of XRD acquisition noise.

(iii) Preparation of the experimental diffraction data for each constituent phase for which spectra are experimentally available.

(iv) Estimation of the optimal set of phase concentrations for each ‘known’ phase by enforcing positivity constraints.

(v) Estimation of the diffraction profiles for the remaining phase constituents.

### 4.1. Background correction

In addition to the sought diffraction peaks, a nonzero background signal is present in all spectra as a result of diffuse scattering and unavoidable experimental imperfections (Cullity, 1956 ▸). It is therefore necessary to estimate this background signal **D**_bg_(2θ, *y*) and subtract it from the raw spectra **D**_raw_(2θ, *y*) prior to any further analysis, 

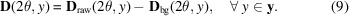

A fourth-order polynomial function is chosen to account for the spectrum background. It should be such that the resulting **D**(2θ, *y*) is always positive after background removal. Thus **D**_bg_ is computed from the minimization of the following cost function 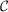 , 

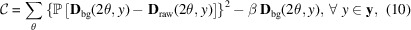

where 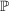 is the ‘positive part’ function [ 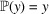 if *y* ≥ 0 and 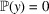 if *y* < 0] and β is a scalar. The ‘penalty’ term 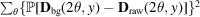 promotes the positivity of **D**(2θ, *y*) so that the background should remain below the raw spectrum, while the second term favours a high background, with a uniform ‘pressure’ β to line up to the minimum values of the spectrum. Because of the presence of XRD noise, the chosen parameter β is to be set so that some negative values are tolerated in the resulting signal **D**(2θ, *y*) (after background correction). In quantitative terms, β is tuned so that the negative values in the residuals have a distribution compatible with the characterized Poisson noise (discussed in the following section). Because of the truncation to negative values, the mean-square value of those negative residuals should be half the noise variance.

In the following, a similar methodology is applied to determine the unknown R-phase spectrum: assuming that at this stage the austenite and martensite spectra are already known, by selecting the appropriate ratio between the pressure and penalty terms, the austenite/martensite XRD contributions can be extracted from the experimental diffraction spectrum while the residual is expected to be ‘positive’. Thus the residual can be further interpreted as the missing R-phase spectrum weighted by its concentrations.

### 4.2. Uncertainty analysis

In order to evaluate the noise associated with XRD analysis, we conducted five scans over a single-phase specimen by varying the XRD acquisition time (*e.g. t* = 5, 10, 15, 20, 25 min). Processing those acquisitions led to the conclusion that the noise is Poisson-like, with a distribution that could not be distinguished from a Gaussian and a variance that varies linearly with the mean count and thus the accumulation time. The motivation of this prior study is to allow an assessment of whether the residual spectrum (difference between measured and estimated spectra) is compatible with the observed noise. In practice, this is done by estimating the differences and normalizing them (for all angles) by the local standard deviation (itself proportional to the square root of the mean signal and acquisition time). Comparing the residual with the noise thus consists of observing that this scaled residual is uniform over all angles and its distribution is a Gaussian of zero mean and unit variance.

### 4.3. Experimental diffraction data for pure phase constituents

In order to collect pure diffraction data while taking potential preferred orientation effects into account, it is recommended to collect diffraction spectra at *N* different spatial coordinates *y* and rearrange them into a matrix form **D**(2θ, *y*) on which the POD analysis is performed: 

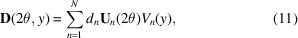

where **U**_*n*_(2θ) represents the *n*th POD angular modes and *V*_*n*_(*y*) is the corresponding spatial amplitude. *d*_*n*_ represents the energetic contribution of the *n*th POD mode to the diffraction matrix as a result of the normalization of each mode, ∥**U**_*n*_∥_θ_ = 1 and ∥*V*_*n*_∥_*y*_ = 1 (the subscript after the norm symbol recalls that these two norms operate in different spaces).

In the following, the discussed test case is such that three phases and only three are expected in this material. With the additional assumption that their orientation is transversely isotropic, it would be expected that no more than three modes are needed to account for the entire set of data. This obviously does not mean that the angular modes **U**_*n*_(2θ) for *n* = 1, 2 or 3 should coincide with the pure-phase spectra, but rather that the linear combinations of these three modes should be sufficient to match any composition of the three phases.

This simple presentation rests on the assumption of a unique type of orientation distribution per phase. Here transverse isotropy is the most likely, but a single orientation or an isotropic distribution would also lead to the same result that no more than three modes are needed to account for all acquired experimental spectra (with the exception of noise which can be considered as an additional mode). In cases where the orientation or orientation distribution is evolving along the loading direction, then more modes should be added with a maximum of three modes per phase, as discussed above (neglecting symmetries that can reduce this number).

It must, however, be emphasized that one of the key properties of POD analysis is that the modes are ordered along a specific hierarchy, according to their relative power contributions (more precisely, the power of the *n*th mode is proportional to its squared eigenvalue, 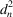 ) in decreasing order. Therefore, the analysis has the potential to proceed with simple assumptions (such as no more than three modes) and test from the residuals (the unexplained data) whether they are compatible with the noise, and hence the initial three-phase assumption is deemed satisfactory, or if the basis should be enriched to account for preferential orientation effects. (Note that, when the residual is within the noise level, this does not mean that the assumptions are correct, but simply that their further refinement cannot be obtained from the currently available noisy XRD data.) Also, because the modes are not the spectra of pure phases, some work is needed to perform a physically meaningful conversion. This is the motivation of the following section.

### 4.4. Positive POD algorithm

For powders, any diffractogram obtained at a given spatial coordinate **D**(2θ, *y*) should be equal to the sum of each pure *i* phase spectrum **S**_*i*_(2θ) weighted by its volume fraction *C*_*i*_(*y*). For austenite, due to its crystalline symmetry (with a body-centred cubic crystalline structure), its experimental XRD spectrum is unique regardless of its crystal orientation. In contrast, we assume that the R and M phases (with a much lower crystalline symmetry than the A phase) are subject to potential variant selection during 1D tensile loading; a different combination of variant selection induces a modulation of different peak intensities for the XRD spectra, and consequently the experimental diffraction patterns for the R and M phases are not unique. 

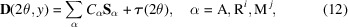




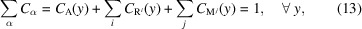

where 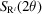 denotes the *i*th R-phase diffraction spectrum, 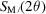 represents the *j*th M-phase diffraction spectrum and 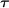 denotes the Poisson noise of the raw signal.

As discussed earlier, the diffraction patterns for A and M variants can be obtained experimentally, whereas the R-phase diffraction profile is as yet unknown. After eliminating the entire signal contribution from all ‘known’ phase constituents 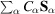 (α denotes the austenite and martensite spectrum), the remaining diffraction signal at any given diffraction angle is expected to remain *positive* for each angle. However, because of the presence of XRD noise, one may tolerate some negative values (as for the case of background signal removal) when consistent with our prior knowledge of its statistical characteristics.

In the following, at each position *y* an optimum set of local phase concentrations 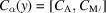 can be obtained through the quadratic minimization of the primary cost function, 

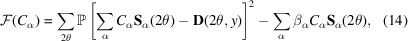

where 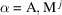 , 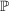 is the positive part function and β_α_ is a positive scalar. Similarly to the background correction, the ‘penalty’ term 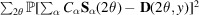 promotes the positivity of the residual signal, and 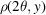 = 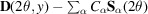 so that the total contribution of all known phase constituents *C*_α_ **S**_α_ should remain below the local diffraction signal. In contrast, the second ‘pressure’ term 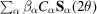 favours a large contribution for each known constituent, which is increased as much as possible (similar to the ‘penalty’ and ‘pressure’ terms introduced for the background).

The minimization of the primary cost function should be carried out under the following convex inequality constraints:

(i) Each phase concentration is expected to be positive, 

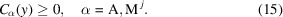




(ii) The sum of all concentrations is expected to be lower than 1, 

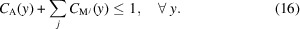




In the following, we propose to group all the above physical inequality constraints on the concentration (15)–(16) into a single matrix form, 

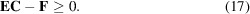




### 4.5. Sub-gradient projection

Finding the optimum set **C** with respect to all positive constraints is a typical convex inequality constrained problem, which consists of minimizing the cost function 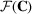 subject to **EC** − **F** ≥ 0. The sub-gradient projection method is frequently used to handle constrained optimization problems. First, the dual cost function (or the augmented Lagrangian) 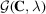 is introduced, 

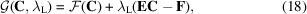

where λ_L_ is the classical augmented Lagrange multiplier to ensure that all physical concentration constraints are fulfilled. The minimization of the dual cost function not only pushes the residual ρ close to 0 while remaining ‘positive’ but also grants the physical admissibility of phase concentrations.

With an initial guess of **C** ^(**k**)^, the sub-gradient can be computed as

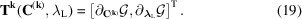

Here **T** ^**k**^(**C** ^(**k**)^, λ_L_) is a positive and monotonic operator. The solution **C** ^(**k**)^ is updated via the sub-gradient projection,

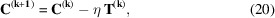

where η is the step length. A relatively small step length is applied to prevent numerical oscillations, η = 1. The value of η could be further optimized for a faster convergence rate. However, because the computation time was not a critical factor, this optimization was not investigated. The sub-gradient projection is repeated until **C** ^(**k**)^ reaches a stationary value, 

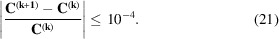

Note that the sub-gradient method is not a descent method; the primal and dual cost function values can (and often do) increase before reaching convergence.

## 5. Positive POD algorithm applied to the case of nickel–titanium alloys

### 5.1. Background correction

**Figure 7:**
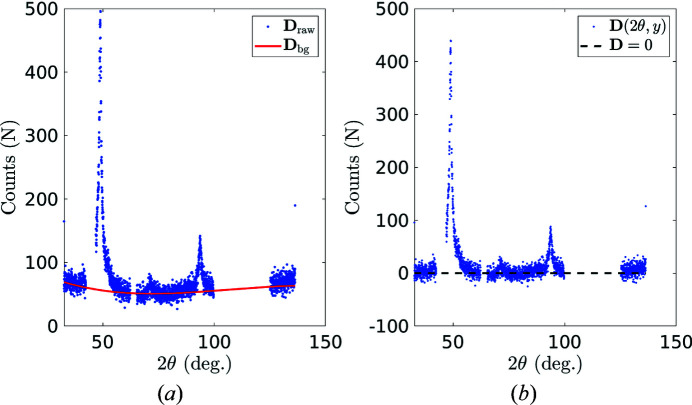
(*a*) The raw diffraction spectrum plotted in blue and the estimated background signal plotted in red. (*b*) The corrected diffraction spectrum after background correction.

An example of the background correction procedure is illustrated in Fig. 7 ▸ using a local spectrum at step 1. The missing channels due to experimental artifacts (*K*β, double absorption by the Fe filter, prism obscuration) make the fourth-order polynomial background signal fitting even more difficult. To overcome this problem, we propose to add additional information from diffraction signals over 2θ ∈ [35°, 45°] ∪ [125°, 135°], which are away from any relevant diffraction peaks; these channels are expected to be centred around zero after background removal. The presence of ‘zero’ channels at the boundary of the spectrum, instead of diffraction peaks, greatly improves the stability of the background correction algorithm.

After choosing the appropriate ratio β = 0.12 between the pushing force and the penalty term [see equation (10)], the background signal is properly estimated (after background correction, for 2θ channels away from {*hkl*} diffraction peaks, the diffraction counts are mostly centred at 0). The differences between the background signals at different spatial positions are very small. Therefore, the average background signal was used in this study to minimize uncertainty. In the following sections, for all four loading stages where XRD scans were carried out, raw diffraction spectra received the same background removal as a pre-processing before launching the p-POD analysis.

### 5.2. Uncertainty analysis

**Figure 8:**
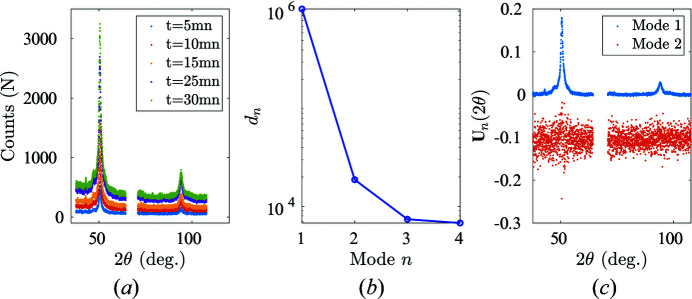
Uncertainty analysis. (*a*) Experimental austenite spectra collected with different acquisition times. (*b*) Eigenvalues of each POD mode ranked in decreasing order. (*c*) Angular shape functions for the first two POD modes. The second mode has been offset by −0.1 for readability.

The uncertainty analysis is carried out on fully austenitic NiTinol at room temperature. Five XRD scans are carried out with corresponding acquisition time intervals *t* = 5, 10, 15, 25, 30 min [Fig. 8 ▸(*a*)], and the differences between these XRD scans are then computed [Fig. 8 ▸(*b*)].

**Figure 9:**
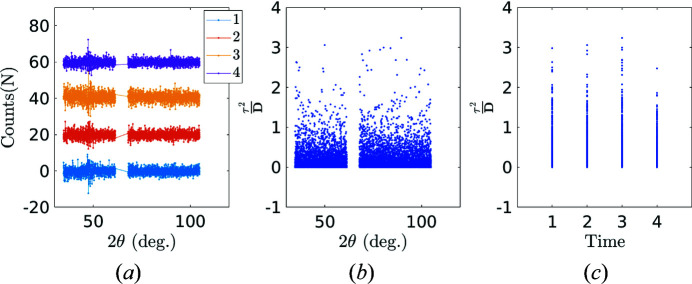
(*a*) The characterized noise follows a Poisson-type distribution. (*b*), (*c*) Angular and temporal stability for re-scaled noise τ^2^/**D** after Anscombe transformation.

The identified noise follows a Poisson-type distribution [the variance scales with the amplitude of the diffraction signals at each channel, see Fig. 9 ▸(*a*)]. When a signal is affected by Poisson noise, its Anscombe transform exhibits a stationary Gaussian noise of uniform variance (Anscombe, 1948 ▸) as soon as the noise amplitude is small compared with the signal. In the present case, the Anscombe transform simply consists of taking the square root of the signal. The re-scaled noise after Anscombe transformation does indeed show the expected variance uniformity over angle and time [see Figs. 9 ▸(*b*) and 9 ▸(*c*), respectively].

### 5.3. Experimental diffraction data collection

#### 5.3.1. Austenite diffraction spectrum

**Figure 10:**
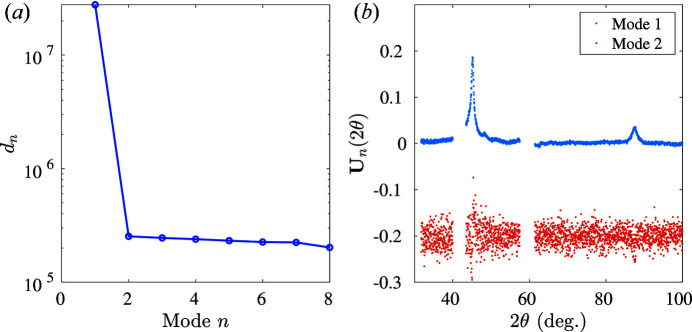
POD analysis applied to the austenite diffraction matrix. (*a*) Eigenvalues of each POD mode ranked in decreasing order. (*b*) The first two POD angular modes.

After thermal heating to transform the 1D NiTinol strip completely into a fully austenite state, the experimental austenite diffraction data can be collected in the stress-free state at room temperature at eight different spatial locations [as shown in Fig. 5 ▸(*a*)]. The POD analysis (Fig. 10 ▸) indicates that, for austenite diffraction profiles, the first POD mode with the highest eigenvalue can be interpreted as the diffraction pattern of austenite, and higher-order POD modes (without any significant peak or structure) are compatible with noise and will be considered as such in the following.

#### 5.3.2. Experimental martensite diffraction spectrum collection

**Figure 11:**
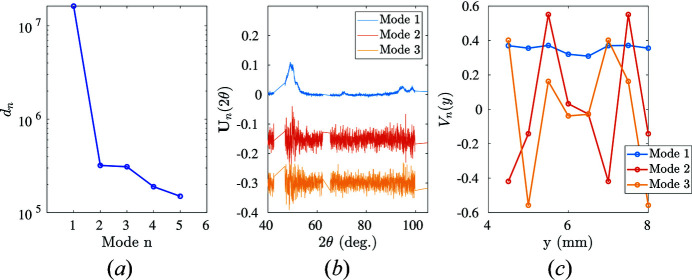
POD analysis applied to experimental martensitic diffraction spectra. (*a*) Eigenvalues of each POD mode ranked in decreasing order. (*b*) The first three POD modes are shown. For clarity, successive modes are offset by −0.15. (*c*) The first three spatial POD modes *V*_*n*_(*y*) as a function of the spatial coordinate *y*.

Experimental martensite diffraction data can be collected when the NiTinol specimen has been fully transformed into martensite [*e.g.* the end of the transformation plateau corresponding to Step 3 in Fig. 4 ▸(*a*)]. In order to take into account potential preferred orientation effects, spectra are acquired at eight different spatial coordinates in the region of interest (*y* = 7, 7.5, 8, 8.5, 9, 9.5, 10 and 10.5 mm). After rearranging the eight different diffraction spectra into a matrix form, a POD analysis is performed (see Fig. 11 ▸). Plotting eigenvalues against POD modes, it is clear that at least two POD modes are needed to describe all spectra faithfully.

The first POD angular mode represents the principal diffraction pattern of martensite. In the range 48 ≤ 2θ ≤ 53°, multiple martensite diffraction peaks contribute: 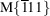 (2θ = 48.3°), M{020} (2θ = 51.4°), M{111} (2θ = 52.4°) and M{012} (2θ = 52.9°). In the range 92 ≤ 2θ ≤ 98°, one finds the characteristic martensite diffraction peaks M{023} (2θ = 93.4°) and M{220} (2θ = 98.9°). Meanwhile, higher-order POD modes, although very noisy, can be seen as the underlying undulation in {*hkl*} intensities due to spatial heterogeneity of ‘martensite detwinning’ across the martensite localization band.

When confronted with the non-uniqueness of the martensite diffraction data due to ‘martensite detwinning’, it is possible to enrich the martensite diffraction profiles by introducing multiple different martensite diffraction patterns. In the present case, two martensite patterns were chosen. The optimum set of two martensitic diffraction spectra [ 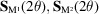 ] should satisfy the constraint of *physical admissibility*, namely, the corresponding phase concentration for each diffraction profile should obey 

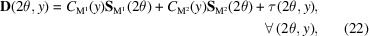




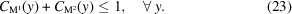

**Figure 12:**
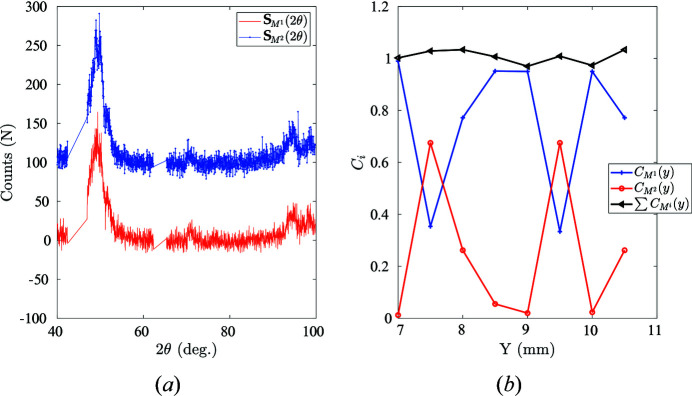
(*a*) Two proposed sets of different martensite diffraction patterns. An offset of 100 counts has been applied at 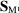 for readability. (*b*) The associated phase concentrations for two different martensite spectra at different spatial coordinates.

One possible example set (this set is not unique) 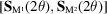 is illustrated in Fig. 12 ▸(*a*). The first experimental martensite diffraction spectrum promotes the M{020} diffraction peak, while the second shows an enhanced intensity for the M{111} peak. The two proposed martensite diffraction spectra consistently represent the entire diffraction matrix, given that the overall sum of these two phase constituents at different spatial coordinates *y* equals almost 1, while each individual concentration remains physically admissible. The reason why the sum of concentrations does not strictly equal 1 may be that two spectra are not enough to give an exhaustive description of the martensite orientations, but the residual data are so few that adding a third phase would make the problem much less robust, and it was decided not to enrich this description.

Note that the experimental profiles for austenite and martensite are now set to the above spectra, **S** = 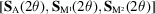 . Hence hereinafter it is possible to estimate the optimum set of phase concentrations for austenite and martensite for Steps 1 and 2 in Fig. 4 ▸(*a*) and eventually to characterize the R-phase diffraction spectrum and its concentration.

### 5.4. Positive POD algorithm applied to scans 1 and 2

**Figure 13:**
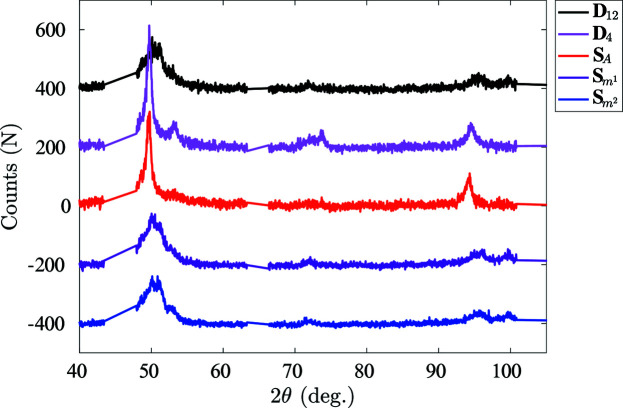
Two investigated local XRD scans and known XRD diffraction data for austenite and martensite. Each spectrum has been offset by 200 counts for readability.

Two local spectra are selected as examples to illustrate the performance of the proposed algorithm. The first spectrum, **D**_12_, is selected for an acquisition positioned inside the transformed bands (with the presence of a strongly preferred orientation effect) while the second spectrum, **D**_4_, is selected at a point which is located at the interface between the austenite matrix and a martensite band (Fig. 13 ▸).

#### 5.4.1. First case: acquisition within the transformed band

**Figure 14:**
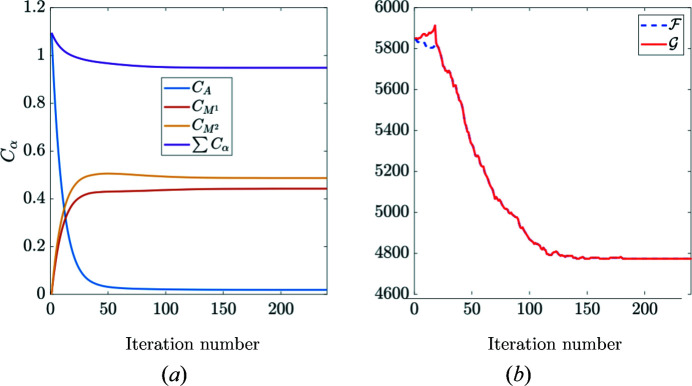
(*a*) Phase concentrations for austenite and martensite constituents. (*b*) The primal/dual cost functions plotted against iteration number.

**Figure 15:**
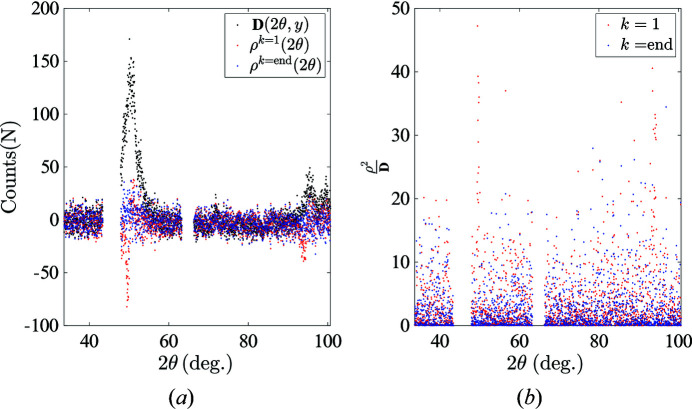
(*a*) A comparison between the raw spectrum, initial residual and final residual. (*b*) Initial and final re-scaled residuals.

At convergence of the p-POD algorithm where both the primal and dual cost functions reach a stationary value [Fig. 14 ▸(*b*)], each constituent concentration is physically admissible and they sum to a value close to 1 [ 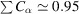 , see Fig. 14 ▸(*a*)]. Furthermore, the final residual almost vanishes when compared with the initial spectrum. When re-scaled by the standard deviation of the XRD noise, it does not show any significant angular dependency. Thus, it can be concluded that the p-POD algorithm can reliably and efficiently account for the preferred orientation effect by introducing physical constraints and two representative martensite diffraction patterns (Fig. 15 ▸).

#### 5.4.2. Second case: acquisition at the interface between the matrix and transformation band

**Figure 16:**
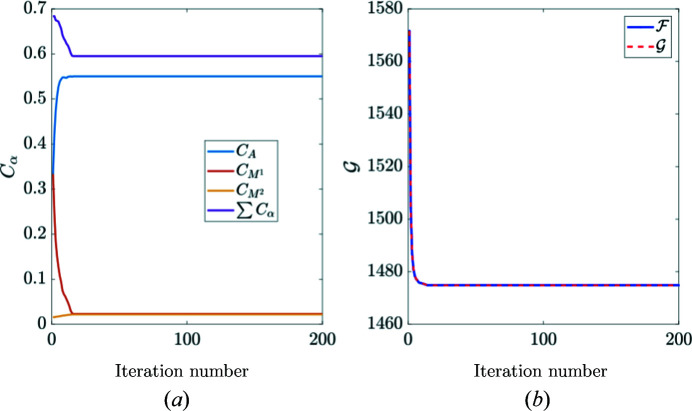
(*a*) Phase concentrations for austenite and martensite. (*b*) Primal/dual cost functions plotted against iteration number.

**Figure 17:**
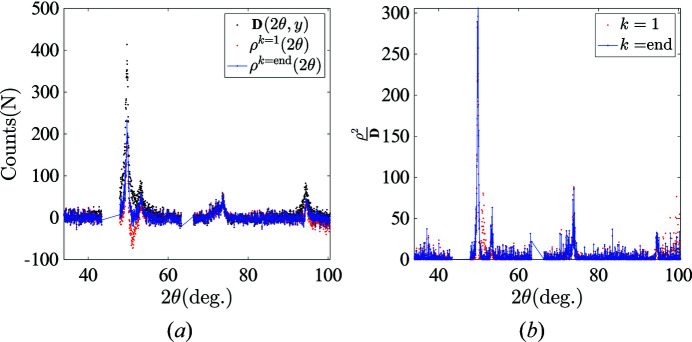
(*a*) A comparison between the raw spectrum, initial residual and final residual. (*b*) Re-scaled residuals.

In the second case, the austenite and martensite concentrations stabilize at convergence (see Fig. 16 ▸) where the sum is well below 1 (0.6). The remaining signal after removing austenite and martensite contributions [Fig. 17 ▸(*a*)] still shows a pronounced angular dependency after Anscombe transformation that cannot be attributed to noise. After considering the locations of the diffraction peaks and their indexing, it is concluded that they correspond to the R phase.

#### 5.4.3. R-phase diffraction pattern reconstruction

**Figure 18:**
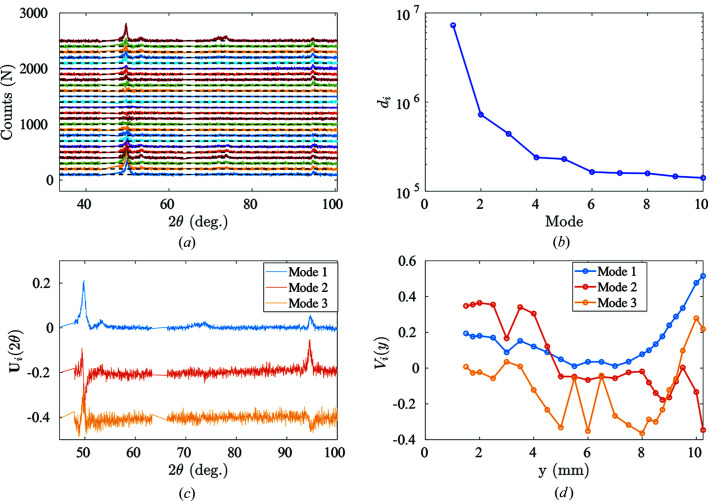
POD analysis applied at ρ. (*a*) The residual diffraction signal after removing austenite and martensite contributions. (*b*) Eigenvalues of each POD mode ranked in decreasing order. (*c*) The first three POD angular modes. (*d*) The first three POD spatial modes.

The p-POD algorithm is applied to the entire set of spectra collected during scan 1. The residual (remaining signal) ρ(2θ, *y*) in the diffraction signal after removal of the diffraction contributions from austenite and martensite is displayed in Fig. 18 ▸(*a*). A POD analysis over ρ(2θ, *y*) shows that at least two POD modes are required to account for the remaining diffraction signal [Fig. 18 ▸(*b*)]. The first POD angular mode corresponds consistently to R-phase powder diffraction peaks, with features such as a double peak ( 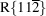 and R{112} at 2θ = 49.3° and 2θ = 49.6°, respectively) or several secondary diffraction peaks (R{202} at 2θ = 52.7° or R{222} at 2θ = 73.6°). Meanwhile, higher-order POD modes can be seen as underlying {*hkl*} intensity undulations due to heterogeneous R-phase detwinning at different spatial positions [Figs. 18 ▸(*c*)–18(*d*) ▸].

**Figure 19:**
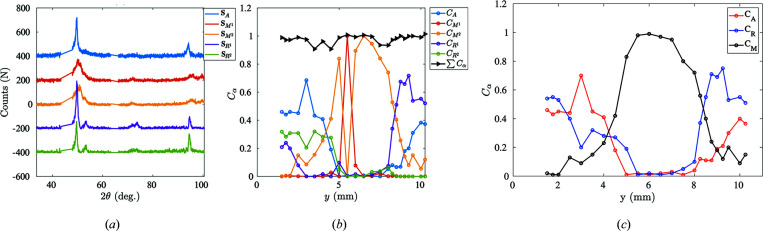
(*a*) The diffraction patterns for austenite, R phase and martensite. Each has been offset by 200 counts for readability. (*b*) The phase concentration for each constituent at different spatial positions. (*c*) The phase concentration for A, R and M phases at different spatial positions.

Similarly to the case of martensite detwinning, it is possible to use two different R-phase diffraction patterns to take into account a preferred orientation effect [see Fig. 19 ▸(*a*)]. The two R-phase diffraction profiles correspond well to the theoretical R-phase diffraction peaks but with different {*hkl*} peak weights. With the complete diffraction pattern, the phase concentrations for each constituent as functions of the scan position are illustrated in Fig. 19 ▸(*b*). All concentrations are always bounded between [0, 1] and their sum never exceeds 1. At several points where the sum of the concentrations is slightly less than 1 (∼0.95), it can be inferred that detwinning introduces multiple R phases and martensite diffraction patterns (not only limited to two). Additional R-phase and martensite diffraction profiles could be further introduced to improve the phase field reconstruction, but the small signal-to-noise ratio becomes limiting.

**Figure 21:**
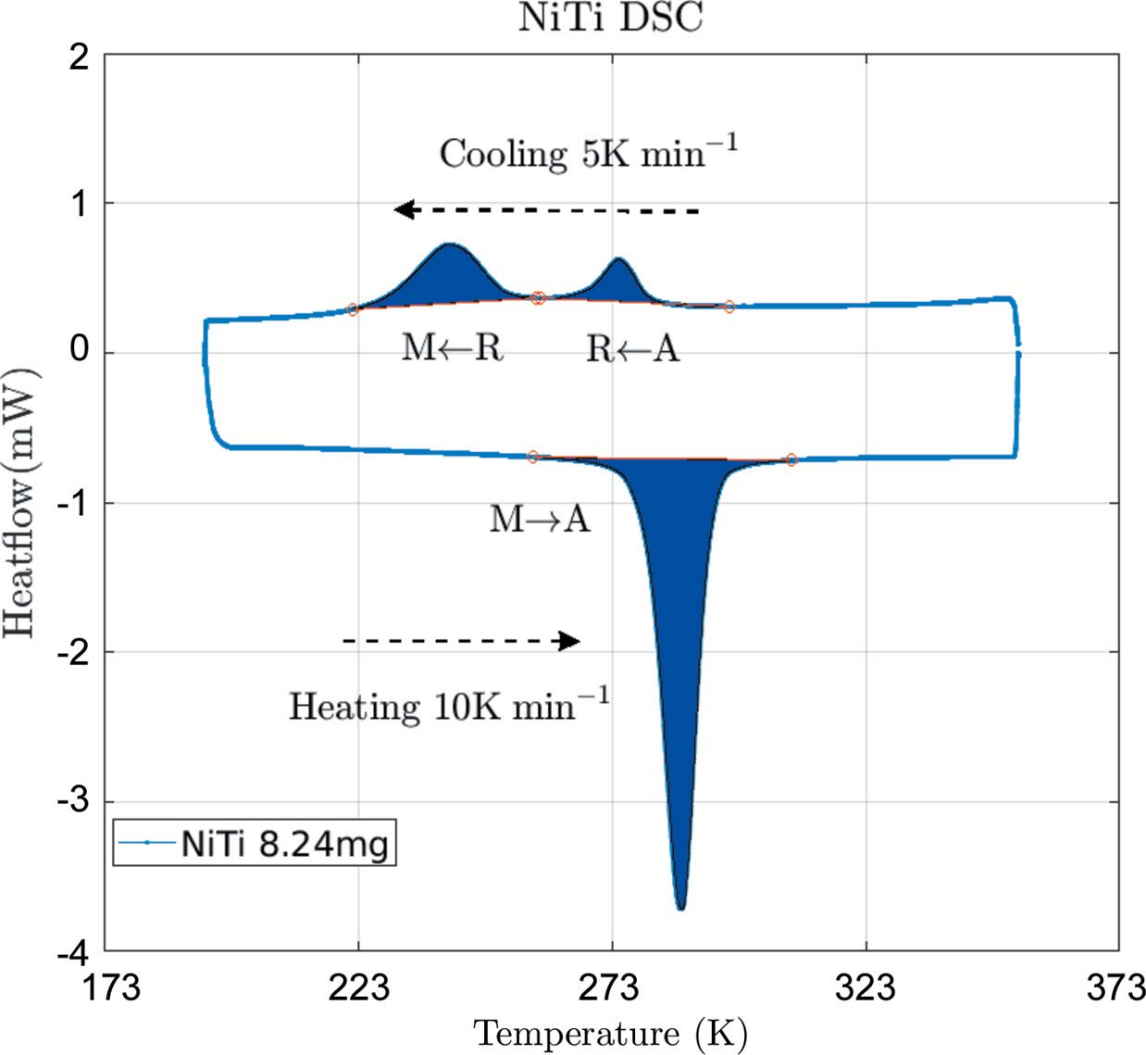
The DSC results for the NiTinol alloy. The two-step phase transformation during thermal loading confirms the presence of the R phase.

Fig. 19 ▸(*c*) shows that the central part of the specimen has transformed into martensite almost completely, whereas the sides are composed of a mixture of A and R phases. Thus it appears that, from pure austenite, the R phase is nucleated under load, coexisting with the austenite, and these two phases eventually turn completely into martensite. This is consistent with the DSC characterization as shown in Fig. 21 (Appendix *B* [App appb]), where A is transformed into M in a two-step process with an R phase as an intermediate phase. The thermal heterogenity during 1D tensile loading could be the reason for this appearance.

## 6. Conclusions

Quantitative XRD analysis remains a great challenge when aiming to obtain a quantitative evaluation of heterogeneous materials. The classical Rietveld method and its generalizations are not adequate when dealing with an incomplete database of diffraction patterns for each phase constituent or undetermined spatially heterogeneous texture distribution for one or several constituents.

When assessing a pure phase with strong spatial heterogeneity in texture distribution, instead of using one unique artificial texture as implemented in the March–Dollase model, the proposed approach uses proper order decomposition to capture the diffraction patterns at different spatial coordinates. This allows for the reconstruction of one or several experimental diffraction patterns, which are much more flexible in accounting for the entire diffraction spectrum including limited orientation modulations.

Moreover, by including inequality constraints into convex minimization, the ‘positive-POD’ algorithm introduced herein can seamlessly remove diffraction contributions from known phase constituents and make it possible to reconstruct diffraction patterns for the unknown constituent afterwards. Additionally, the accuracy of the reconstructed unknown constituent can be verified by powder diffraction indexing. This algorithm can be considered as a step forward compared with the March model. Its extreme versatility appears to make it a promising tool in quantitative XRD analysis for complex heterogeneous materials.

## Figures and Tables

**Figure 20:**
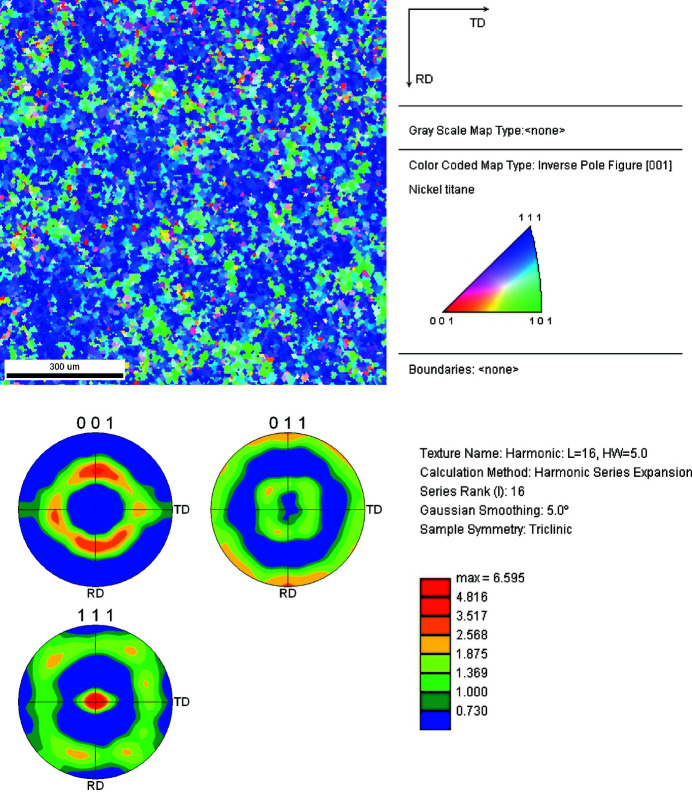
(Top) Inverse pole figure and (bottom) pole figures for the equiatomic NiTinol alloy.

## Appendix A Crystal orientation of equiatomic NiTinol

As shown in Fig. 20 ▸, the equiatomic NiTinol exhibits a very pronounced anisotropy in its crystal lattice orientation, as a result of its lamination. The texture orientation is along the 〈111〉 direction, underlining its transverse isotropic texture.

## Appendix B Differential scanning calorimetry

The results of DSC measurement of equiatomic NiTinol are shown in Fig. 21 ▸. The presence of the R phase is manifest along the cooling curve as an intermediate step between austenite and martensite.
